# SMSSVD: SubMatrix Selection Singular Value Decomposition

**DOI:** 10.1093/bioinformatics/bty566

**Published:** 2018-07-13

**Authors:** Rasmus Henningsson, Magnus Fontes

**Affiliations:** 1 The Centre for Mathematical Sciences, Lund University, Lund, Sweden; 2 The International Group for Data Analysis, Institut Pasteur, Paris, France; 3 The Center for Genomic Medicine, Rigshospitalet, Copenhagen, Denmark; 4 Persimune, The Centre of Excellence for Personalized Medicine, Copenhagen, Denmark

## Abstract

**Motivation:**

High throughput biomedical measurements normally capture multiple overlaid biologically relevant signals and often also signals representing different types of technical artefacts like e.g. batch effects. Signal identification and decomposition are accordingly main objectives in statistical biomedical modeling and data analysis. Existing methods, aimed at signal reconstruction and deconvolution, in general, are either supervised, contain parameters that need to be estimated or present other types of ad hoc features. We here introduce SubMatrix Selection Singular Value Decomposition (SMSSVD), a parameter-free unsupervised signal decomposition and dimension reduction method, designed to reduce noise, adaptively for each low-rank-signal in a given data matrix, and represent the signals in the data in a way that enable unbiased exploratory analysis and reconstruction of multiple overlaid signals, including identifying groups of variables that drive different signals.

**Results:**

The SMSSVD method produces a denoised signal decomposition from a given data matrix. It also guarantees orthogonality between signal components in a straightforward manner and it is designed to make automation possible. We illustrate SMSSVD by applying it to several real and synthetic datasets and compare its performance to golden standard methods like PCA (Principal Component Analysis) and SPC (Sparse Principal Components, using Lasso constraints). The SMSSVD is computationally efficient and despite being a parameter-free method, in general, outperforms existing statistical learning methods.

**Availability and implementation:**

A Julia implementation of SMSSVD is openly available on GitHub (https://github.com/rasmushenningsson/SubMatrixSelectionSVD.jl).

**Supplementary information:**

Supplementary data are available at *Bioinformatics* online.

## 1 Introduction

High throughput biomedical measurements, by design, normally capture multiple overlaid biologically relevant signals, but often also signals representing different types of biological and technical artefacts. The artefacts can arise due to random differences in types of cells analyzed for different samples, synchronized cell cycles, sample handling in the lab and biased measurement errors to name just a few. Since these represent unknown properties of the samples, they cannot be controlled for and thus are best seen as (structured) noise. On top of this, there is in general white noise adding uncertainty to the data.

There exist different methods aimed at signal reconstruction and deconvolution of the resulting high dimensional and complex datasets, but these methods almost always contain parameters that need to be estimated or present other types of ad hoc features. Developed specifically for Omics data and more particularly gene expression data such methods include the gene shaving method (Hastie *et al.*, 2000), tree harvesting (Hastie *et al.*, 2001), supervised principal components (Bair and Tibshirani, 2004) and amplified marginal eigenvector regression (Ding and McDonald, 2017). They employ widely different strategies to deal with the ubiquitous *P *≫* N* (many more variables than samples) problem in omics data. Gene Shaving uses the first principal component to iteratively guide variable selection towards progressively smaller nested subsets of correlated genes with large variances. An optimal subset size is then chosen using the ‘gap statistic’, a measure of how much better the subset is than what is expected by random chance. To find additional subsets (signals), each gene is first projected onto the orthogonal complement of the average gene in the current subset, and the whole process is repeated.

We here introduce SubMatrix Selection Singular Value Decomposition (SMSSVD), a parameter-free unsupervised dimension reduction technique primarily designed to reduce noise, adaptively for each low-rank-signal in a data matrix, and represent the data in a way that enable unbiased exploratory analysis and reconstruction of the multiple overlaid signals, including finding the variables that drive the different signals.

Our first observation for the theoretical foundation of SMSSVD is that the SVD of a linear map restricted to a hyperplane (linear subspace) share many properties with the SVD of the corresponding unrestricted linear map. Using this we show that, by iteratively choosing orthogonal hyperplanes based on criteria for optimal variable selection and concatenating the decompositions, we can construct a denoised decomposition of the data matrix. The SMSSVD method guarantees orthogonality between components in a straightforward manner and coincide with the SVD if no variable selection is applied. We illustrate the SMSSVD by applying it to several real and synthetic datasets and compare its performance to golden standard methods for unsupervised exploratory analysis: Classical PCA (Principal Component Analysis) (Hotelling, 1933) and the lasso or elastic net based methods like SPC (Sparse Principal Components) (Witten *et al.*, 2009). Just like PCA and SPC, SMSSVD is intended for use in wide range of situations, and no assumptions specific to gene expression analysis are made in the derivation of the method. The SMSSVD is computationally efficient and despite being a parameter-free method, in general, it outperforms or equals the performance of the golden standard methods. A Julia implementation of SMSSVD is openly available on GitHub.

## 2 Materials and methods

**Fig. 1.**
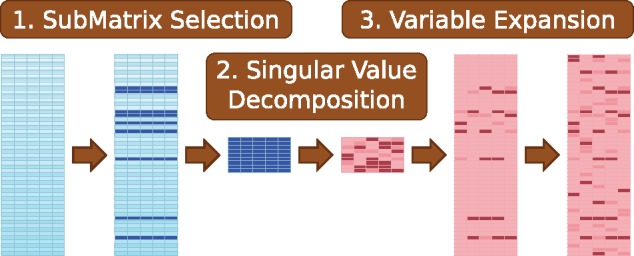
Overview of the SMSSVD algorithm. It starts from a *P *×* N* data matrix with *P* variables and *N* samples (the leftmost matrix in the figure). 1. A subset of the variables are selected, creating a smaller data matrix. 2. A low-rank representation of the new matrix is computed using SVD. 3. The representation is expanded to the full set of variables, producing a low-rank representation of the strongest signal in the dataset. 4. (Not depicted.) The signal is removed from the original data matrix and the process is repeated to find more signals

SubMatrix Selection Singular Value Decomposition (SMSSVD), is outlined in Figure 1. The basic idea is simple: when extracting a signal from a data matrix, we work only with a subset of the variables, chosen such that variables that are non-informative (i.e. noisy) are avoided. This is a common strategy. What makes SMSSVD stand out is that the extracted signal is then expanded, in a straightforward and mathematically sound way, to the full set of variables. That gives SMSSVD several desirable properties. *1. Interpretability* in terms of the of full set of variables. *2. Iterability*—multiple signals can be extracted by repeating the procedure, the variable selection can be done separately for each signal, and it’s possible for variables to contribute to multiple signals. *3. Orthogonality*, meaning that different dimensions can be interpreted separately from each other. *4.* It is *parameter-free*, i.e. no tuning is required when applying SMSSVD to a dataset.

Below, we will describe the mathematical foundation for SMSSVD. Proofs and some technical details can be found in the Supplementary Materials—the focus here will be on interpreting the mathematics behind SMSSVD. Throughout the text, *X* will denote a *P *×* N* data matrix, where *P* is the number of variables and *N* the number of samples.

The variable selection step is critical for SMSSVD, as it provides the basis for the adaptive noise reduction mechanism of SMSSVD. (In fact, if variable selection is omitted, the SMSSVD of *X* will coincide with the SVD of *X*.) The Projection Score (Fontes and Soneson, 2011) provides a natural optimality criterion for variable selection. It is a measure of how informative a specific variable subset is, when constructing a rank *d* approximation of a data matrix. As a rough approximation, we can expect that variables with higher variance are less influenced by noise, in high throughput biological data. Given a variance filtering threshold, we can construct a subset of variables by keeping precisely those variables that have variance above the threshold. Thus, by optimizing the Projection Score jointly over the variance filtering threshold and the dimension, we get both an optimal variable subset and a simple dimension estimate *d* of the signal that was captured.

Performing SVD on the post variable selection $\tilde{P}\times N$ matrix (let’s call it $\tilde{X}$) provides a lot of information. If we keep only the *d* largest singular values, we get a low-rank representation $\tilde{U}\tilde{\Sigma }{\tilde{V}}^{T}$ of $\tilde{X}$, where $\tilde{U}\in R^{\tilde{P}\times d}$, $\tilde{\Sigma }\in R^{d\times d}$ and $\tilde{V}\in R^{N\times d}$. The *d* columns of $\tilde{V}$ contain the (unscaled) sample coordinates used to create a PCA (Principal Component Analysis) plot of $\tilde{X}$. The $\tilde{U}$ matrix does however only contain the variable information of $\tilde{P}$ out of the *P* variables. Interestingly, the matrices $\tilde{U}$ and $\tilde{V}$ are tightly connected. If we know $\tilde{V}$, then it turns out that $\tilde{U}$ can be recovered, since $\tilde{X}\tilde{V}=\tilde{U}\tilde{\Sigma }$ (a well-known property of the SVD). In Theorem 2.1, we generalize this idea and show how we can expand the $\tilde{V}$ constructed from $\tilde{X}$ to a low-rank representation of *X* itself. The columns of the matrix $\tilde{V}$ defines a *d*-dimensional subspace Π of the sample space $R^{N}$. The variable expansion works by considering *X* (viewed as a linear map) restricted to Π. In this manner, we can move the sample representation created from the smaller matrix $\tilde{X}$ to a variable representation of the original matrix *X*.

Our first theorem will describe the relationship between the SVD of *X* restricted to some subspace Π and the matrix *X*. The second theorem builds on the first and shows how the variable selection affect the final result.

Theorem 2.1 (Decomposition Theorem). *Let*$X{|}_{\Pi }:\Pi \to X(\Pi )$*be the restriction of a linear map*$X:R^{N}\to R^{P}$*to a d-dimensional subspace*$\Pi \subset R^{N}$*such that*Π⊥kerX*. Furthermore, let*$U\Sigma V^{T}=\sum_{i=1}^{d}\sigma_{i}U_{\cdot i}V_{\cdot i}^{T}$*be the singular value decomposition of*$X{|}_{\Pi }$*. Then*

1. $V_{\cdot i}⊥\ker\;X,\;\forall i$.
2. $U_{\cdot i}⊥\text{coker}\;X,\;\forall i$.
3. *XV* = *U*Σ.
4. $U^{T}X=\Sigma V^{T}+U^{T}X(I-VV^{T})$.
5. $(I-UU^{T})X(I-VV^{T})=(I-UU^{T})X$.
6. $\text{rank}\left(X\right)=d+\text{rank}\left((I-UU^{T})X\right)$.

Remark. In the statement of the theorem we consider all vectors to belong to the full-dimensional spaces. In particular, we extend all vectors in subspaces of the full spaces with zero in the orthogonal complements.

Proof. See Supplementary Materials. □

Note that *VV^T^* is the orthogonal projection on Π and *UU^T^* is the orthogonal projection on *X*(Π). If Π is spanned by the right singular vectors corresponding to the *d* largest singular values of *X*, then *U*Σ*V^T^* is the truncated SVD which by the *Eckhart-Young Theorem* is the closest rank *d* matrix to *X* in Frobenius and Spectral norms. Furthermore, if $\Pi ={\left(\ker X\right)}^{⊥}$, then *d* = rank *X* and *U*Σ*V^T^* is the SVD of *X* (without expanding *U* and *V* to orthonormal matrices). That is, for some particular choices of Π, *U*Σ*V^T^* corresponds directly to the SVD. But even when this is not the case, many important properties that hold for the (truncated) SVD of *X* are retained regardless of how the subspace Π is chosen. Note however that for the SVD, property *4* is symmetric to property *3*, i.e. *U^T^X*  =  Σ*V^T^*, but the residual *U^T^X*(*I*– *VV^T^*) is nonzero in general.

To adaptively reduce noise, Π must depend on *X*. By optimizing over the Projection Score, we can select a subset of the variables that are likely to be less influenced by noise. This is a special case of choosing Π after performing a linear transform of the variables.

Theorem 2.2 (Selection-Expansion Theorem). *Take a linear map*$S:R^{L}\to R^{P}$*and an integer d such that rank*$\;S^{T}X\geq d$*and let*$\tilde{U}\tilde{\Sigma }{\tilde{V}}^{T}$*be the rank d truncated SVD of S^T^X. Furthermore let Π be the subspace spanned by the columns of*$\tilde{V}$*and let UΣV^T^ be the SVD of*$X{|}_{\Pi }$. *Then*

1. Π⊥ker X.
2. $S^{T}U\Sigma V^{T}=\tilde{U}\tilde{\Sigma }{\tilde{V}}^{T}$.
3. $\left\{V_{\cdot 1},V_{\cdot 2},\ldots ,V_{\cdot d}\right\}$*and*$\left\{{\tilde{V}}_{\cdot 1},{\tilde{V}}_{\cdot 2},\ldots ,{\tilde{V}}_{\cdot d}\right\}$*are orthonormal bases of* Π.
4. $\left\{S^{T}U_{\cdot 1},S^{T}U_{\cdot 2},\ldots ,S^{T}U_{\cdot d}\right\}$*and*$\left\{{\tilde{U}}_{\cdot 1},{\tilde{U}}_{\cdot 2},\ldots ,{\tilde{U}}_{\cdot d}\right\}$*are bases of*$S^{T}X(\Pi )$.
5. $||\Sigma |{|}_{F}\geq \frac{||\tilde{\Sigma }|{|}_{F}}{||S|{|}_{2}}$.
6. $U^{T}X=\Sigma V^{T}+U^{T}(I-SS^{T})X(I-VV^{T})$.

Proof. See Supplementary Materials. □

Corollary 2.1. *If S^T^S* = *I*, *then*$||\Sigma |{|}_{F}\geq ||\tilde{\Sigma }|{|}_{F}$.

The properties in Theorem 2.2 show that the Selection-Expansion procedure works as expected. First, property *1* simply states that Theorem 2.1 can be applied. Property *2* tells us that variable selection is the inverse operation of variable expansion, in the sense that if we apply variable selection to the low-rank *P *×* N* matrix *U*Σ*V^T^*, we retrieve the unexpanded low-rank $\tilde{P}\times N$ matrix $\tilde{U}\tilde{\Sigma }{\tilde{V}}^{T}$. Thus, the variable expansion expands the representation the full set of variables, while leaving the selected variables intact. From property *3*, it is clear that the sample representation is in essence the same for expanded matrix as for the smaller one, they can only differ by rotation/reflection. It follows that PCA-style biplots based on the small or expanded matrices will look identical (up to rotation/reflection of the whole plot)—apart from the obvious difference that the biplot of the expanded matrix will show loadings of all variables and not only the selected ones. We also note that the residual term $U^{T}(I-SS^{T})X(I-VV^{T})$ in property *6* [cf. *U^T^X*(*I* – *VV^T^*) in Theorem 2.1, property *4*] is here shown to only depend on the non-selected variables, again what we would expect. Finally, Corollary 2.1 explains that the singular values of the expanded matrix will always be greater than or equal to those of the smaller matrix (in Frobenius norm).

Another way to interpret *S* is that *SS^T^* defines a (possibly degenerate) inner product on the sample space, which is used to find Π. To see this, let *d* = rank *S^T^X* so that $\tilde{U}\tilde{\Sigma }{\tilde{V}}^{T}=S^{T}X$ and $K:=X^{T}SS^{T}X=\tilde{V}{\tilde{\Sigma }}^{2}{\tilde{V}}^{T}$, showing the well-known result that $\tilde{V}{\tilde{\Sigma }}^{2}{\tilde{V}}^{T}$ is an eigendecomposition of *K*, where $K_{ij}=\langle x_{i},x_{j}\rangle :=X_{\cdot i}^{T}SS^{T}X_{\cdot j}$ is the inner product of sample *i* and *j*. This naturally extends to kernel PCA, where *K* is defined by taking scalar products after an (implicit) mapping to a higher-dimensional space. Any method that results in a low-dimensional sample space representation $\tilde{V}$ can indeed be used, since Π is spanned by the columns of $\tilde{V}$ by definition. We will not pursue these extensions here.

We are now ready to state the SMSSVD algorithm that was outlined in Figure 1. Let $X_{1}:=X$ and repeat the following steps for k=1,2,…

1. **Selection:** Optimize over the Projection Score to find the optimal variable selection matrix *S_k_* and signal dimension *d_k_* for the matrix *X_k_*.
2. **SVD:** Let Π_*k*_ be the subspace spanned by the columns of ${\tilde{V}}_{k}$ in the rank *d_k_* truncated SVD of $S_{k}^{T}X_{k}$.
3. **Expansion:** Compute $U_{k}\Sigma_{k}V_{k}^{T}$ from $X_{k}{|}_{\Pi_{k}}$.
4. **Signal Removal:** Let $X_{k+1}:=(I-U_{k}U_{k}^{T})X_{k}$.

The iterations can continue as long as *X_k_* is nonzero or until some other stopping criteria is met. Finally, the signals are concatenated:

$$\begin{array}{c} U\Sigma V^{T}:=\left(\begin{array}{cccc} U_{1} & U_{2} & \ldots & U_{n} \end{array}\right)\left(\begin{array}{cccc} \Sigma_{1} & & & \\ & \Sigma_{2} & & \\ & & \ddots & \\ & & & \Sigma_{n} \end{array}\right)\left(\begin{array}{c} V_{1}^{T}\\ V_{2}^{T}\\ \vdots \\ V_{n}^{T} \end{array}\right)\\ =\sum_{k=1}^{n}U_{k}\Sigma_{k}V_{k}^{T}, \end{array}$$

where *U*Σ*V^T^* is the SMSSVD of *X*, a noise-reduced (and low-rank) version of *X*.

SMSSVD is designed to keep as many properties of SVD as possible, while still reducing the influence from noise for datasets with many variables. The representation itself, *U*Σ*V^T^*, is strikingly similar to the SVD, and the parts of the decomposition can be interpreted in the same ways as for SVD, thus allowing for similar visualization and downstream analysis.

One of the reasons that the SVD is ubiquitously used is that both *U* and *V* have orthonormal columns, which greatly aids interpretation since different effects can be separated from each other. This is also true for the SMSSVD. First, orthonormality between columns within each *U_k_* and *V_k_* follow immediately from the definition ($U_{k}\Sigma_{k}V_{k}^{T}$ is the SVD of $X_{k}{|}_{\Pi_{k}}$). Second, the ‘Signal Removal’ step enforces orthogonality between signals. It makes sure that the columns of *U_k_* are in coker *X_l_* for all *l *>* k*, and orthogonality follows since *U_l_* is guaranteed to be orthogonal to coker *X_l_* (Theorem 2.1, property *3*). Similarly, since $X_{k+1}=(I-U_{k}U_{k}^{T})X_{k}=(I-U_{k}U_{k}^{T})X_{k}(I-V_{k}V_{k}^{T})$, the same holds true for the *V_k’_*s.

In SVD, the diagonal elements of Σ are ordered in descending order. For SMSSVD, this is true within each Σ_*k*_, but not necessarily between signals. But, in practice we do not expect them to deviate much from being in descending order, since the SMSSVD algorithm is designed to pick the strongest signals from the data first.

The ‘Signal Removal’ step reduces the rank of the data matrix by *d_k_*, which is the rank of signal *k*, by Theorem 2.1, property *6*. This implies that $\text{rank}U\Sigma V^{T}=\text{rank}\;X$ if the iterations are run all the way until *X_k_* = 0, which is what we would expect. But, in contrast to SVD, *U*Σ*V^T^* ≠ *X* in general, due to the noise removal.

## 3 Results

The performance of SMSSVD is evaluated in comparison to SVD and SPC (Sparse Principal Components), a method similar to SVD, but with an additional lasso (*L*_1_) constraint to achieve sparsity (Witten *et al.*, 2009). The methods are evaluated both for real data using four gene expression datasets and for synthetic data where the ground truth is known. All comparisons are done with the same number of dimensions in the different models, i.e. SMSSVD and SPC are run until the target dimension has been reached and the SVD is truncated to use the top *d* singular values.

### 3.1 Gene expression data

**Fig. 2.**
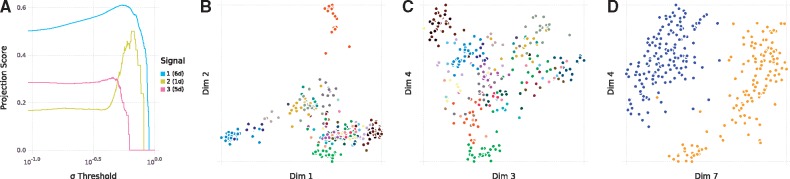
SMSSVD of the TCGA dataset. **A**. Projection Scores for each signal, with threshold for variable filtering on the x-axis, i.e. only variables with higher standard deviation than the threshold are included. **B–D**. Sample plots. B and C are colored by tumor type (see Supplementary Materials for details). Some examples are Liver hepatocellular carcinoma (orange, top of B), Brain Lower Grade Glioma (light blue, bottom left of B), Ovarian serous cystadenocarcinoma (light green, bottom of B), Rectum adenocarcinoma (dark purple, top left of C) and Colon adenocarcinoma (brown, top left of C). In D, the samples are colored by gender, Female (yellow) and Male (blue)

A proof of concept of SMSSVD is shown in Figure 2, using gene expression data from TCGA (The Cancer Genome Atlas) (Weinstein *et al.*, 2013). The dataset was downloaded from recount2 (Fu *et al.*, 2018), the first 300 samples with the annotation ‘cgc_case_tumor_status’ set to ‘WITH TUMOR’ were used and the samples were labeled according to the ‘gdc_cases_tissue_source_site_project’ annotation, with 30 different tumor types. Normalization was done using the Variance Stabilizing Transformation (VST) (Anders and Huber, 2010; Love *et al.*, 2014). Panel A displays the Projection Score for the signals found by SMSSVD, as a function of the variance filtering threshold. The strongest signal (6d, 991 variables selected) dominates the dataset with a high Projection Score for a wide range of variance filtering values, but still with a clear peak, showing that we get a more robust signal after variance filtering. In panels B and C, we see how this signal captures gene expression differences between tumor types in the dataset. In contrast, the second signal (1d, 8 variables selected), has a more well-defined peak, but cannot be found without variance filtering. As evident in panel D, it corresponds to gender, a signal not captured by e.g. SVD. The ability to find both kinds of signals, in an unsupervised, unbiased, manner, showcases SMSSVD. The third signal (5d, 161 variables selected), is a bit harder to capture, but we do still see a single peak in the projection score plot. It also corresponds to differences between tumor types. A more complete view can be seen in Supplementary Figures S1 and S2, showing the first 12 dimensions for SMSSVD and SVD respectively.

We also tried SMSSVD on three other gene expression datasets, two openly available with microarray data and one based on RNA-Seq available upon request from the original authors. Gene expression microarray profiles from a study of breast cancer (Chin *et al.*, 2006) was previously used to evaluate SPC (Witten *et al.*, 2009), but in contrast to their analysis, we use all 118 samples and all 22215 genes. Each sample was labeled as one of five breast cancer subtypes: ‘basal-like’, ‘luminal A’, ‘luminal B’, ‘ERBB2’ and ‘normal breast-like’. In a study of pediatric Acute Lymphoblastic Leukemia (ALL), gene expression profiles were measured for 132 diagnostic samples (Ross *et al.*, 2003). The samples were labeled by prognostic leukemia subtype [‘TEL-AML1’, ‘BCR-ABL’, ‘MLL’, ‘Hyperdiploid (>50)’, ‘E2A-PBX1’, ‘T-ALL’ and ‘Other’]. Our final dataset is from another pediatric ALL study, where gene expression profiling was done from RNA-Seq data for 195 samples (Lilljebjörn *et al.*, 2016). The samples were aligned with Tophat2 (Kim *et al.*, 2013) and gene expression levels were normalized by TMM (Robinson and Oshlack, 2010) and log-transformed. Only genes with a support of at least 10 reads in at least 2 samples were kept. The annotated subtypes in this dataset were ‘BCR-ABL1’, ‘ETV6-RUNX1’, ‘High hyperdiploid’, ‘MLL’, ‘TCF3-PBX1’ and ‘Other’. Here, ‘Other’ is a very diverse group containing everything that did not fit in first five categories. We thus present results without this group included (the results with ‘Other’ included can be found in Supplementary Fig. S3).

**Fig. 3.**
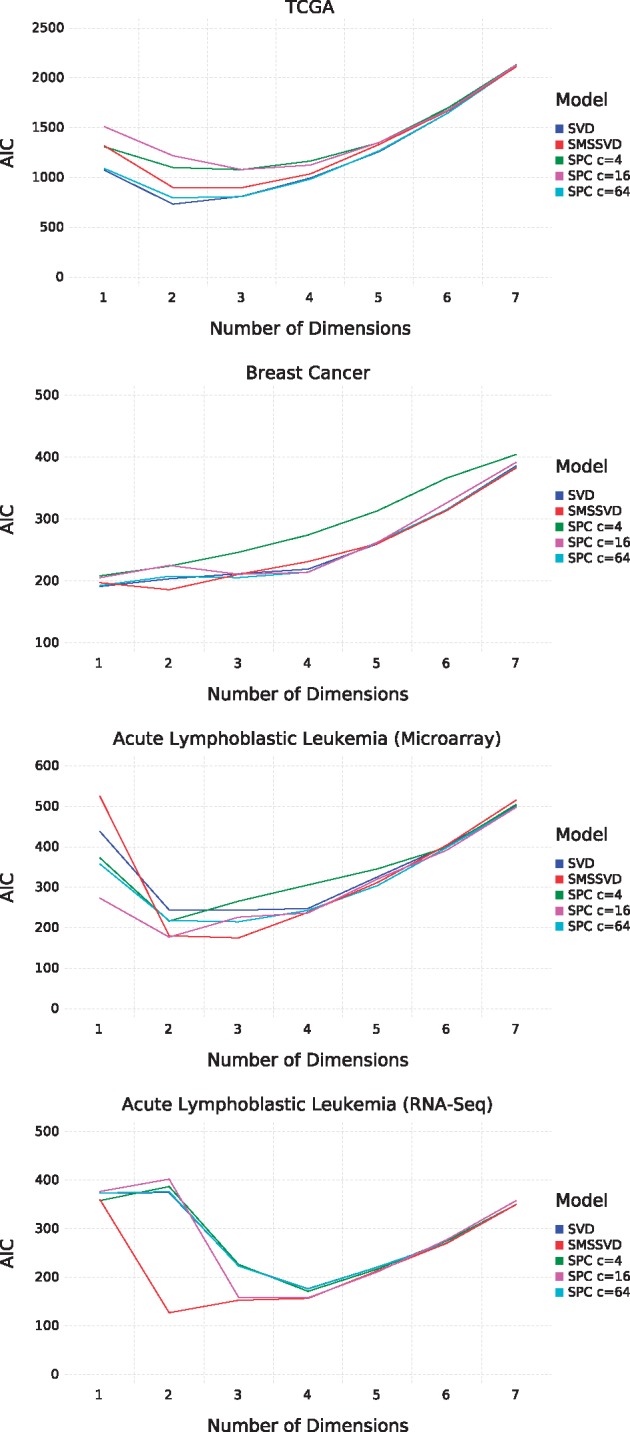
Evaluation of SMSSVD on different datasets, based on AIC scores when fitting a Gaussian Mixture Model to the (sub)types. From top to bottom: TCGA dataset, Breast Cancer, Acute Lymphoblastic Leukemia (Microarray), Acute Lymphoblastic Leukemia (RNA-Seq)

The ability to extract relevant information from the gene expression datasets was evaluated for each model by how well they could explain the (sub)types, using the Akaike Information Criterion (AIC) for model scoring. Given the low-dimensional sample representations from SMSSVD, SVD or SPC (for different values of the sparsity parameter, *c*), a Gaussian Mixture Model was constructed by fitting one Multivariate Gaussian per subtype. The class priors were chosen proportional to the size of each subtype. The loglikelihood l:=log P(x|θ,M), where **x** are the subtype labels, *M* is the model and *θ* a vector of *k* fitted model parameters is used to compute the AIC = 2*k* – 2 *l*. Scoring models in this way is not a universal method to determine a ‘best’ model. It relies on annotations that are unlikely to capture all the structure in the data and in addition different subtypes cannot always be assumed to follow Gaussian distributions, even though the approximation can be believed to be reasonably accurate since the (sub)type characteristics are comprised of many smaller effects. We do however believe that it is helpful to have a rough measure of the biological relevance of the models, even though it is impossible to provide a ground truth. Figure 3 displays the AIC scores for the different models as a function of the model dimension. SMSSVD generally performs better than SVD. Comparison with SPC is trickier, since the performance of SPC is determined by the sparsity parameter *c* and there is no simple objective way to choose *c*. However, SMSSVD compares well with SPC regardless of the value of the parameter.

Scree plots are often used to delineate signal from noise, by removing components after the ‘knee’ in the plot. Supplementary Figure S4 displays scree plots of the four datasets in Figure 3. It is non-trivial and subjective to determine where the knee is, for the TCGA dataset, it can be argued to be at component 2, 4 or 6. SMSSVD avoids these problems by instead automatically determining signal dimension by optimizing the Projection Score. Furthermore, the components after the ‘knee’ can still contain important information. SMSSVD finds biologically relevant structure in all later components (7–12), relating samples either to gender or tumor type, see Supplementary Figure S1. This is also true to some extent for SVD (Supplementary Fig. S2).

### 3.2 Synthetic data

SMSSVD decomposes a matrix observed in noisy conditions as a series of orthogonal low-rank signals. The aim is to get a stable representation of the samples and then recover as much as possible of the variables, even for signals that are heavily corrupted by noise. To evaluate SMSSVD, we synthetically create a series of low-rank signals *Y_k_* that are orthogonal (i.e. $Y_{i}^{T}Y_{j}=0$ and $Y_{i}Y_{j}^{T}=0$ for *i *≠* j*) and that has a chosen level of sparsity on the variable side and try to recover the individual *Y_k_*’s from the observed matrix $X:=\sum_{k}Y_{k}+\varepsilon$ where *ε* is a matrix and $\varepsilon_{ij}∼N(0,\sigma_{ij})$. To measure how well SMSSVD recovers the signals from the data, we look at each signal separately, considering only variables where the signal has support. Let err(*k*) be the reconstruction error of signal *k*,

$$\text{err}(k):=||R_{k}^{T}(Y_{k}-{\hat{Y}}_{k})|{|}_{F},$$

where ${\hat{Y}}_{k}$ is the reconstructed signal and *R_k_* is defined such that multiplying with $R_{k}^{T}$ from the left selects the variables (rows) where *Y_k_* is nonzero.

While SMSSVD is designed to find *d*-dimensional signals (${\hat{Y}}_{k}:=U_{k}\Sigma_{k}V_{k}^{T}$), the same is not true for SVD and SPC. To test the ability to find the signals, rather than the ability to find them in the right order, the components are reordered using a algorithm that tries to minimize the total error by greedily matching the rank 1 matrices from the decomposition to signals *Y_k_*, always picking the match that lowers the total error the most. The number of rank 1 matrices matched to each signal *Y_k_* is equal to rank *Y_k_*. Note that with no noise present, SVD is guaranteed to always find the optimal decomposition.

**Fig. 4.**
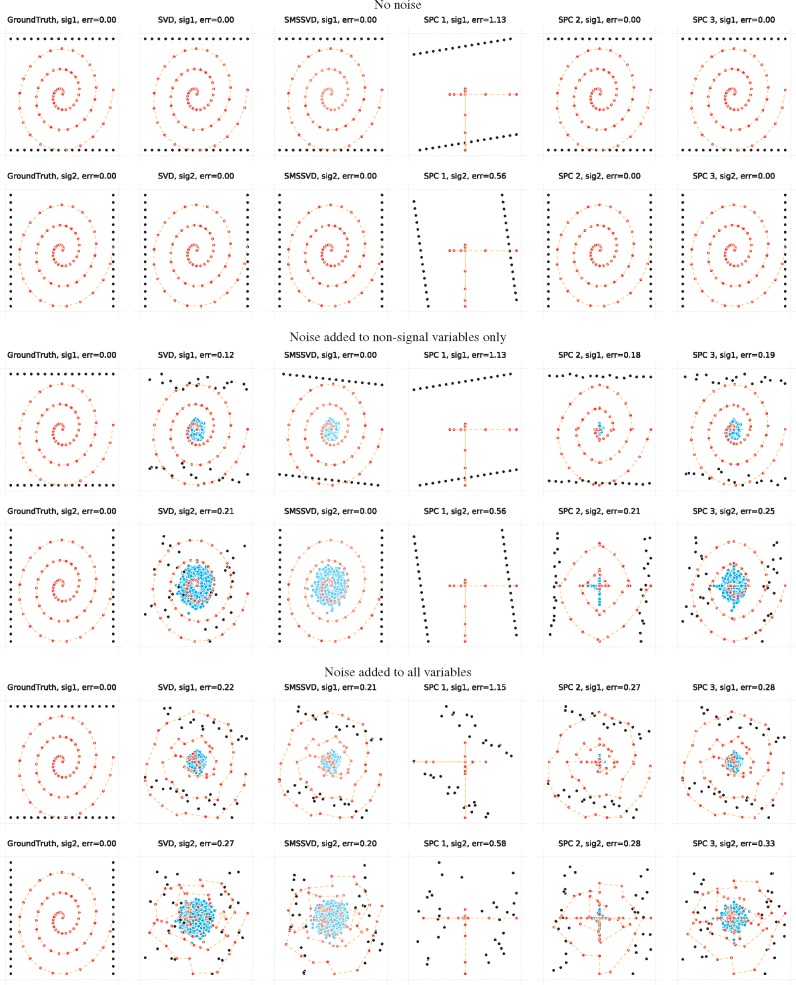
Two 2*d* signals with non-overlapping support for the variables are shown for no noise (Upper two rows), noise added to non-signal variables only (Middle two rows) and for noise added to all variables (Lower two rows). The reconstruction of the first signal is shown in the upper row and for the second signal in the lower row in each set. Different columns correspond to different methods, where SPC ‘1’, ‘2’ and ‘3’ have regularization penalties of *c *=* *2, 8 and 32 respectively, controlling the degree of sparsity. Samples are black, variables where the signal has support are red and other variables are blue. The variables in the support are connected with dashed lines, only to make it easier to spot how the variables are influenced by noise. For SMSSVD, variables selected by optimal variance filtering are shown in full color and other variables are shown in a whiter tone. Samples and variables are both scaled to fill the axes in each biplot. 32 samples and 5000 variables were used, of which each signal had support in 64 variables and the rest had noise only

The biplots in Figure 4 illustrate how SMSSVD works and how the signal reconstructions compare to other methods. If there is no noise, perfect decompositions are achieved by all methods apart from SPC with a high degree of sparsity. An artificial example where the noise is only added to the non-signal variables highlights that SMSSVD can still perfectly reconstruct both samples and signal variables, whereas the other methods display significant defects. Finally, when all variables are affected by noise, SMSSVD still get the best results.

**Fig. 5.**
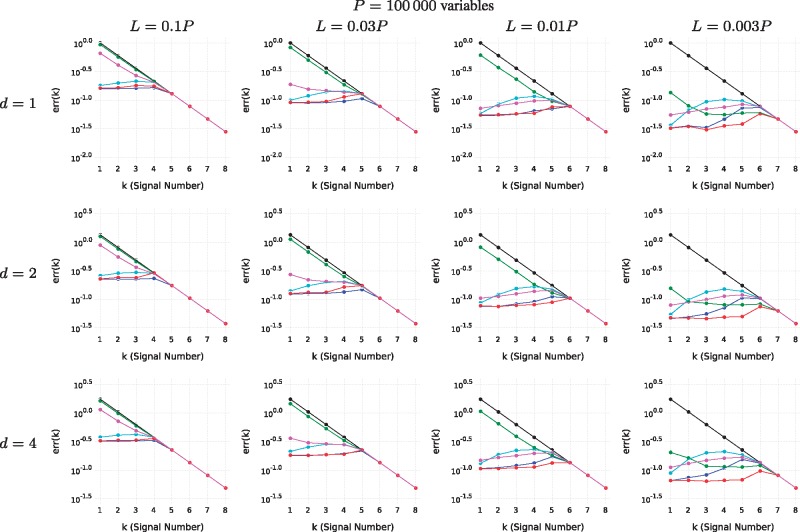
The reconstruction error, err(*k*), is shown for different conditions. The signal strength $||Y_{k}|{|}_{F}$ (black) is shown for scale. The methods are: SVD (blue), SMSSVD (red) and SPC (green, magenta, cyan) with decreasing degree of sparsity (regularization parameters $c=0.04\sqrt{P},\;c=0.12\sqrt{P}$ and $c=0.36\sqrt{P}$ respectively). No errors larger than the signal strength are displayed as that indicates that a different signal has been found

**Fig. 6.**
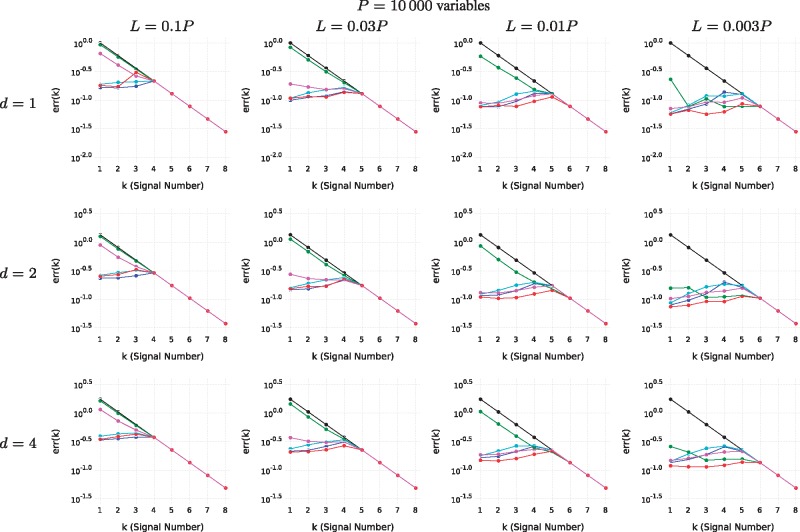
The reconstruction error, err(*k*), is shown for different conditions. The signal strength $||Y_{k}|{|}_{F}$ (black) is shown for scale. The methods are: SVD (blue), SMSSVD (red) and SPC (green, magenta, cyan) with decreasing degree of sparsity (regularization parameters $c=0.04\sqrt{P},\;c=0.12\sqrt{P}$ and $c=0.36\sqrt{P}$ respectively). No errors larger than the signal strength are displayed as that indicates that a different signal has been found

Next, we created several datasets for a variety of conditions based on the parameters *N *=* *100: Number of samples, *P*: Number of variables, *L*: Number of variables in the support of each signal, *K *=* *8: number of signals and *d*: the rank of each signal. For each signal, we randomize matrices *U_k_* and *V_k_*, choose a diagonal matrix Σ_*k*_ and let $Y_{k}:=U_{k}\Sigma_{k}V_{k}^{T}$. For both *V_k_* and *U_k_*, each new column is created by sampling a vector of i.i.d. Gaussian random variables and projecting onto the orthogonal complement of the subspace spanned by previous columns (in current and previous signals). For *U_k_*, we only consider the subspace spanned by *L* randomly selected variables. The result is then expanded by inserting zeros for the other *P* – *L* variables. To complete the signal, let the *i*’th diagonal element of Σ_*k*_, ${\left(\Sigma_{k}\right)}_{ii}:=0.6^{k-1}0.9^{i-1}$, such that there is a decline in the power between signals and within components of each signal. Finally, i.i.d. Gaussian noise is added to the data matrix. Figures 5, 6 and Supplementary Figure S5 show test results for datasets randomized in this way for different sets of parameters. SMSSVD is the only method that performs well over the whole set of parameters. The only situation where SMSSVD is consistently outperformed is by SVD for large *L*, and it is by a narrow margin. SMSSVD performs particularly well, in comparison to the other methods, in the difficult cases when the signal to noise ratio is low. SPC performance clearly depends on the regularization parameter which must be chosen differently in different situations. However, despite being a parameter-free method, SMSSVD outperforms SPC in most cases.

### 3.3 Computation time

SMSSVD is more computationally intensive than SVD or SPC, since it relies on bootstrapping to compute the Projection Score. However, very few bootstrap iterations are required in general since the variances of the eigenvalues of the randomized matrices tend to be very small. Furthermore, since no parameter tuning is needed, it is often sufficient to run SMSSVD only once. Execution times for the datasets in Figure 3 are shown in Table 1.

**Table 1.** Execution times on an Intel Core i7-4720HQ CPU @ 2.6 GHz.

	SVD	SPC^a^ (*c*=)			SMSSVD	
Dataset		4	16	64	10 iter.	100 iter.
TCGA	1.61s	7.4s	8.5s	9.2s	75s	396s
Breast Cancer	0.28s	1.7s	1.8s	2.5s	13s	49s
ALL (Microarray)	0.35s	2.4s	2.6s	3.2s	21s	77s
ALL (RNA-Seq)	0.08s	0.6s	0.5s	0.5s	9s	30s

*Note*: 7 dimensions were computed in all cases.

a Our implementation of SPC.

## 4 Discussion

We have presented SMSSVD, a dimension reduction technique designed for complex datasets with multiple overlaid signals observed in noisy conditions. When compared to other methods, over a wide range of conditions, SMSSVD performs equally well or better. SMSSVD excels in situations where *P *≫* N* (many more variables than samples) and most of the variables just contribute noise, a very common situation for high throughput biological data. As a parameter-free method, SMSSVD requires no assumptions to be made of the level of sparsity. Indeed, SMSSVD can handle different signals within the same dataset that exhibit very different levels of sparsity. Being parameter-free also makes SMSSVD suitable for automated pipelines, where few assumptions can be made about the data.

A common strategy when analyzing high dimensional data is to first apply PCA (SVD) to reduce the dimension to an intermediate number, high enough to give an accurate representation of the dataset, but low enough to get rid of some noise and to speed up downstream computations [see e.g. (Maaten and Hinton, 2008)]. We argue that since SMSSVD can recover multiple overlaid signals and adaptively reduce the noise affecting each signal so that even signals with a lower signal to noise ratio can be found, it is very useful in this situation.

Our unique contribution is that we first solve a more suitable dimension reduction problem for robustly finding signals in a dataset corrupted by noise and then map the result back to the original variables. We also show how this combination of steps gives SMSSVD many desirable properties, related to the SVD of both the full data matrix and of the smaller matrix from the variable selection step. Orthogonality between components is one of the cornerstones of SVD, but it is often difficult to satisfy the orthogonality conditions when other factors are taken into account. SPC does for instance give orthogonality for samples, but not for variables and the average genes of each subset in gene shaving are ‘reasonably’ uncorrelated. For SMSSVD, orthogonality follows immediately from the construction, simplifying interpretation and subsequent analysis steps. Theorem 2.2, property *2* highlights that the variables retained in the variable selection step are unaffected when the solution is expanded to the full set of variables. Hence, we can naturally view each signal from the point of view of the selected variables, or using all variables.

The variable selection step in the SMSSVD algorithm can be chosen freely. For exploratory analysis, optimizing the Projection Score based on variance filtering is a natural and unbiased choice. Another option is to use Projection Score for response related filtering, e.g. ranking the variables by the absolute value of the *t*-statistic when performing a *t*-test between two groups of samples. The algorithm also has verbatim support for variable weighting, by choosing the *S* matrix as a diagonal matrix with a weight for each variable. Clearly this is a generalization of variable selection.

Kernel PCA, SPC and other methods that give low-dimensional sample representations, but where the variable information is (partially) lost, can also be extended by SMSSVD (relying on Theorem 2.1 only), if a linear representation in the original variables can be considered meaningful. Apart from retrieving a variable-side representation, the SMSSVD algorithm also makes it possible to find multiple overlapping signals, by applying the dimension reduction method of interest as the first step of each SMSSVD iteration.

SMSSVD was evaluated on several gene expression and synthetic datasets and performs very well in comparison to golden standard methods for unsupervised exploratory analysis. The SMSSVD model is not limited to gene expression datasets, but is intended for any datasets where at least some of the signals can be expected to have support in a limited number of variables, a very common situation for high throughput biological data. We have in fact already applied SMSSVD in the study of viral quasispecies, when modeling viral populations as distributions over sequence space (Henningsson *et al.*, 2018).

## Supplementary Material

Supplementary Materials
