# Supplementary material for: SMSSVD: SubMatrix Selection Singular Value Decomposition
Source: Bioinformatics. 2018 Jul 13;35(3):478–86. doi: 10.1093/bioinformatics/bty566 (PMC6361234; doi:10.1093/bioinformatics/bty566)
Supplement: Supplementary Materials [file bty566_supplementary_materials.pdf]

Supplementary Materials

SMSSVD – SubMatrix Selection Singular Value Decomposition

Proofs and Comments

Theorem 1 (Decomposition Theorem)**.** *Let* $X\big|_\Pi : \Pi \to X(\Pi)$ *be the restriction of a linear map* $X : \mathbb{R}^N \to \mathbb{R}^P$ *to a* d*-dimensional subspace* $\Pi \subset \mathbb{R}^N$ *such that* $\Pi \perp \ker X$*. Furthermore, let* $U\Sigma V^T = \sum_{i=1}^d \sigma_i U_{\cdot i} V_{\cdot i}^T$ *be the singular value decomposition of* $X\big|_\Pi$*. Then*

1. $V_{\cdot i} \perp \ker X,\ \forall i.$
2. $U_{\cdot i} \perp \operatorname{coker} X,\ \forall i.$
3. $XV = U\Sigma.$
4. $U^T X = \Sigma V^T + U^T X(I - VV^T).$
5. $(I - UU^T)X(I - VV^T) = (I - UU^T)X.$
6. $\operatorname{rank}(X) = d + \operatorname{rank}\big((I - UU^T)X\big).$

Remark. *In the statement of the theorem and in the proof below, we consider all vectors to belong to the full-dimensional spaces. In particular, we extend all vectors in subspaces of the full spaces with zero in the orthogonal complements.*

Proof. 1. The columns of V are an orthonormal basis of Π and thus orthogonal to $\ker X$. *2.* The columns of U are an orthonormal basis of $X(\Pi)$ and $X(\Pi) \perp \operatorname{coker} X$. *3.* $XV = X\big|_\Pi V = U\Sigma V^T V = U\Sigma$. *4.* Using *3* we get

$$\begin{aligned} U^T X &= U^T X V V^T + U^T X(I - VV^T) \\ &= \Sigma V^T + U^T X(I - VV^T). \end{aligned}$$

5. The statement follows from $(I - UU^T)XV = (I - UU^T)U\Sigma = \mathbf{0}$, where we have used that $U^T U = I$. *6.* Let $Y \coloneqq X(\Pi)$ and $Z \coloneqq \operatorname{im} X / X(\Pi)$ be the parts of the decomposition $\operatorname{im} X = Y \oplus Z$, which is possible since $Y \subset \operatorname{im} X$. The linear map $(I - UU^T)$ is orthogonal projection onto $X(\Pi)^\perp$ and thus maps $Y \to 0$ and $Z \to Z$. Since $\operatorname{rank} A = \dim(\operatorname{im} A)$, it follows immediately that $\operatorname{rank}(I - UU^T)X = \dim Z$ and that $\operatorname{rank} X = \dim Y + \dim Z = d + \dim Z$. □

Theorem 2 (Selection-Expansion Theorem)**.** *Take a linear map* $S : \mathbb{R}^L \to \mathbb{R}^P$ *and an integer* d *such that* $\operatorname{rank} S^T X \geq d$ *and let* $\tilde{U}\tilde{\Sigma}\tilde{V}^T$ *be the rank* d *truncated SVD of* $S^T X$*. Furthermore let* Π *be the subspace spanned by the columns of* $\tilde{V}$ *and let* $U\Sigma V^T$ *be the SVD of* $X\big|_\Pi$*. Then*

1. $\Pi \perp \ker X.$
2. $S^T U\Sigma V^T = \tilde{U}\tilde{\Sigma}\tilde{V}^T.$
3. $\{V_{\cdot 1}, V_{\cdot 2}, \ldots, V_{\cdot d}\}$ *and* $\{\tilde{V}_{\cdot 1}, \tilde{V}_{\cdot 2}, \ldots, \tilde{V}_{\cdot d}\}$ *are orthonormal bases of* Π.
4. $\{S^T U_{\cdot 1}, S^T U_{\cdot 2}, \ldots, S^T U_{\cdot d}\}$ *and* $\{\tilde{U}_{\cdot 1}, \tilde{U}_{\cdot 2}, \ldots, \tilde{U}_{\cdot d}\}$ *are bases of* $S^T X(\Pi)$.
5. $\|\Sigma\|_F \geq \frac{\|\tilde{\Sigma}\|_F}{\|S\|_2}.$

6. $U^T X = \Sigma V^T + U^T(I - SS^T)X(I - VV^T)$.

Proof. 1. The columns of $\tilde{V}$ are orthogonal to $\ker S^T X \supset \ker X$. *2.* $S^T U \Sigma V^T = S^T X\big|_\Pi = (S^T X)\big|_\Pi = \tilde{U}\tilde{\Sigma}\tilde{V}^T$. *3.* Follows immediately from the definitions. *4.* $\{\tilde{U}_{\cdot i}\}_{i=1}^d$ is a basis of $S^T X(\Pi)$. By property *2*, $\tilde{U} = S^T U \Sigma V^T \tilde{V} \tilde{\Sigma}^{-1}$, showing that $\{S^T U_{\cdot i}\}_{i=1}^d$ span $\{\tilde{U}_{\cdot i}\}_{i=1}^d$. Finally, since U and $\tilde{U}$ have the same rank, $\{U_{\cdot i}\}_{i=1}^d$ is also a basis of $S^T X(\Pi)$. *5.* For general matrices A and B, consider A acting on each column of B. We get

$$\|AB\|_F^2 = \sum_i \|AB_{\cdot i}\|_2^2 \leq \sum_i \|A\|_2^2 \|B_{\cdot i}\|_2^2 = \|A\|_2^2 \|B\|_F^2.$$

The result now follows from property *2*, with $A = S^T$ and $B = U\Sigma V^T$, since $\|AB\|_F = \|\tilde{U}\tilde{\Sigma}\tilde{V}^T\|_F = \|\tilde{\Sigma}\|_F$ and $\|B\|_F = \|\Sigma\|_F$. *6.* From Theorem 1, property *4*, we get $U^T X = \Sigma V^T + U^T X(I - VV^T)$. It remains to show that $U^T SS^T X(I - VV^T) = \mathbf{0}$. By property *4*, there exists a matrix Z such that $S^T U = \tilde{U} Z$ and

$$\begin{aligned} U^T SS^T X(I - VV^T) &= Z^T \tilde{U}^T S^T X(I - VV^T) \\ &= Z^T \tilde{\Sigma}\tilde{V}^T (I - \tilde{V}\tilde{V}^T) = \mathbf{0}, \end{aligned}$$

where $VV^T = \tilde{V}\tilde{V}^T$ because of property *3*. □

Even if the SMSSVD algorithm is run until $X_k = 0$, $U\Sigma V^T \neq X$ in general, with equality iff the residual $U_k^T X_k (I - V_k V_k^T) = 0$ for all k. Indeed, if $U\Sigma V^T = X$, then the SMSSVD of X coincides with the SVD of X (up to permutation of the singular values and corresponding singular vectors). If instead $U\Sigma V^T \neq X$, let's consider the residual term $U^T X - \Sigma V^T$, which corresponds to what is removed by the noise reduction. By the 'Signal Removal' step in the SMSSVD algorithm,

$$X_n = (I - U_{n-1}U_{n-1}^T)(I - U_{n-2}U_{n-2}^T) \cdots (I - U_1 U_1^T)X.$$

Hence, $U_k^T X = U_k^T X_k$ by Theorem 1, property *4*, and the residual takes the form

$$U^T X - \Sigma V^T = \begin{pmatrix} U_1^T (I - S_1 S_1^T) X_1 (I - V_1 V_1^T) \\ U_2^T (I - S_2 S_2^T) X_2 (I - V_2 V_2^T) \\ \vdots \\ U_n^T (I - S_n S_n^T) X_n (I - V_n V_n^T) \end{pmatrix}.$$

Supplementary Figures

Color key for Supplementary Figures 1 and 2:
Below the diagonal, samples are colored by the annotation 'xml_gender', Female and Male.
Above the diagonal, samples are colored by the annotation 'gdc_cases_tissue_source_site_project': Mesothelioma, Kidney renal clear cell carcinoma, Rectum adenocarcinoma, Bladder Urothelial Carcinoma, Adrenocortical carcinoma, Lung squamous cell carcinoma, Pheochromocytoma and Paraganglioma, Kidney Chromophobe, Uterine Corpus Endometrial Carcinoma, Sarcoma, Uveal Melanoma, Head and Neck squamous cell carcinoma, Thyroid carcinoma, Colon adenocarcinoma, Stomach adenocarcinoma, Skin Cutaneous Melanoma, Kidney renal papillary cell carcinoma, Cervical squamous cell carcinoma and endocervical adenocarcinoma, Lymphoid Neoplasm Diffuse Large B-cell Lymphoma, Breast invasive carcinoma, Uterine Carcinosarcoma, Esophageal carcinoma, Prostate adenocarcinoma, Lung adenocarcinoma, Cholangiocarcinoma, Liver hepatocellular carcinoma, Ovarian serous cystadenocarcinoma, Pancreatic adenocarcinoma, Brain Lower Grade Glioma and Glioblastoma multiforme.

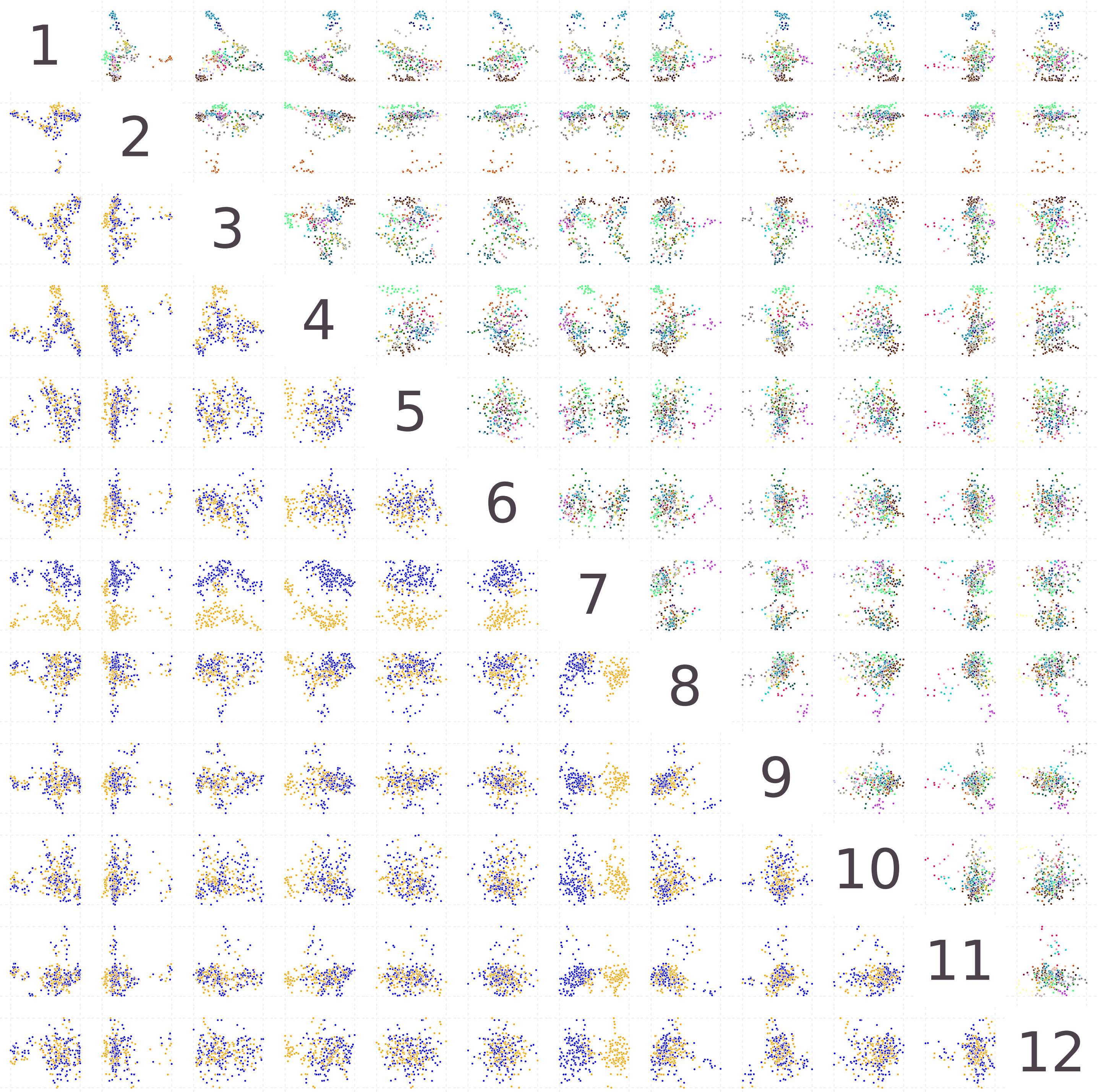

Supplementary Figure 1: SMSSVD of the TCGA data set. Below the diagonal, samples are colored by 'xml_gender', and above the diagonal, samples are colored by 'gdc_cases_tissue_source_site_project'. (Color key given above.)

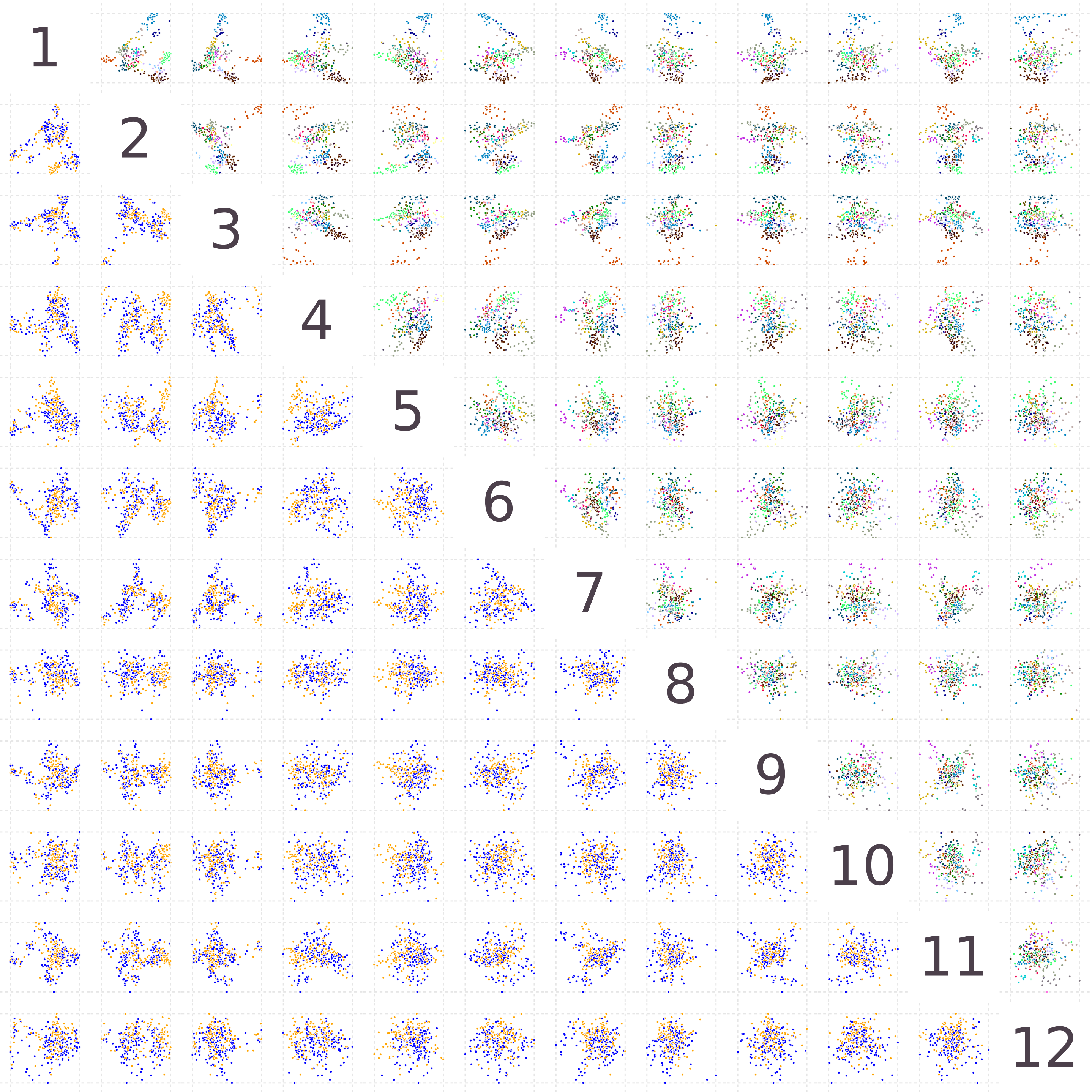

Supplementary Figure 2: SVD of the TCGA data set. Below the diagonal, samples are colored by 'xml_gender', and above the diagonal, samples are colored by 'gdc_cases_tissue_source_site_project'. (Color key given above.)

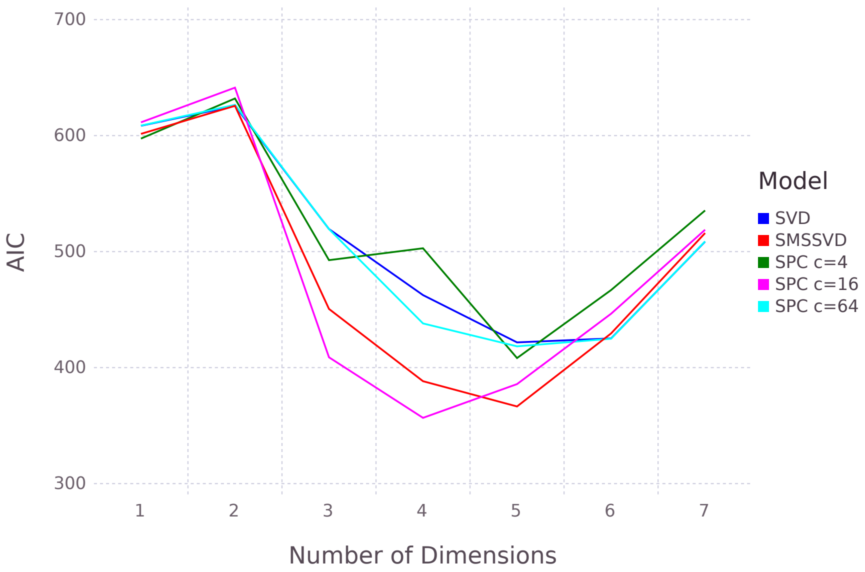

Supplementary Figure 3: Evaluation of SMSSVD on the Acute Lymphoblastic Leukemia (RNA-Seq) data set with subtype ‘Other’ included.

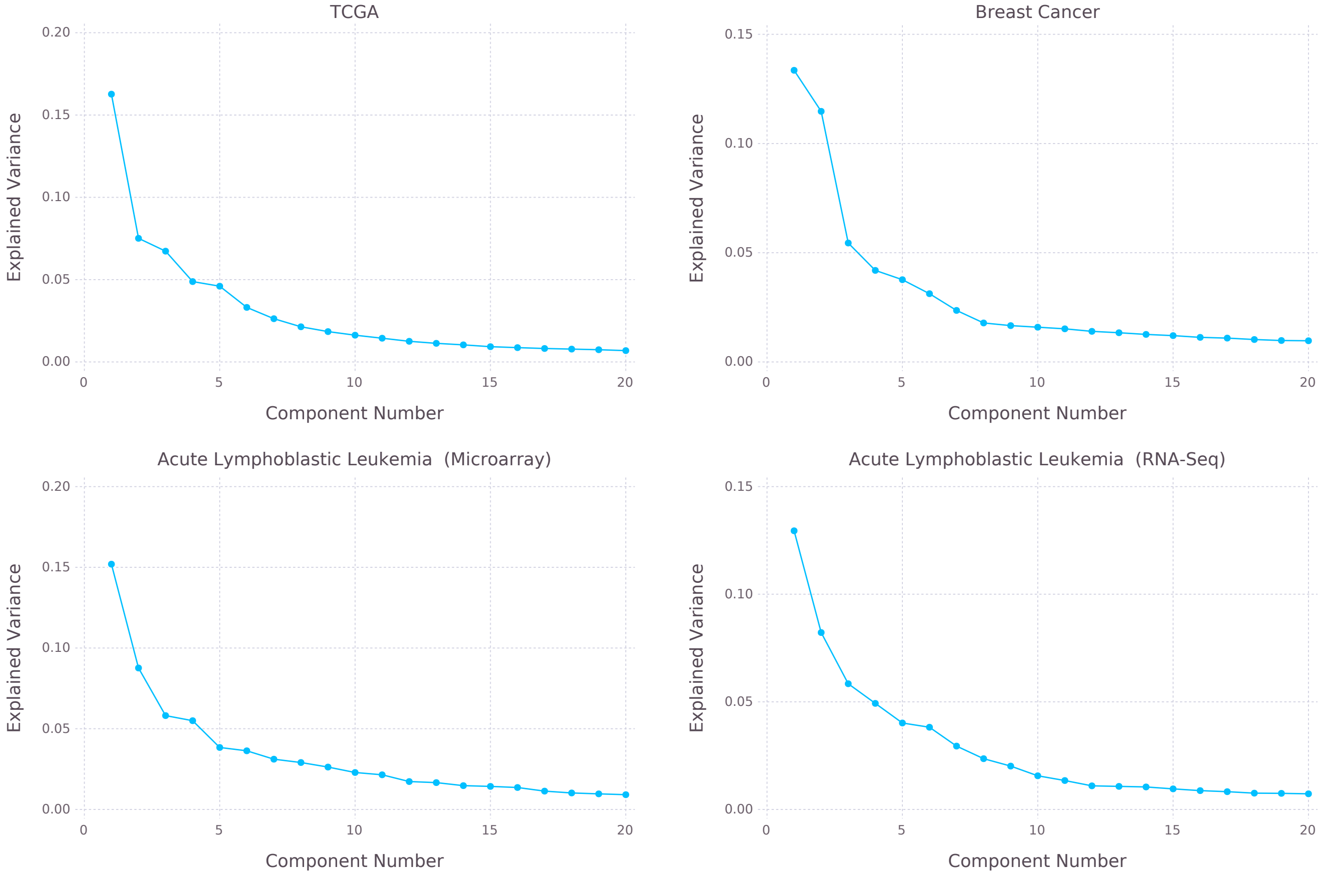

Supplementary Figure 4: Scree plots for the data sets in Figure 3.

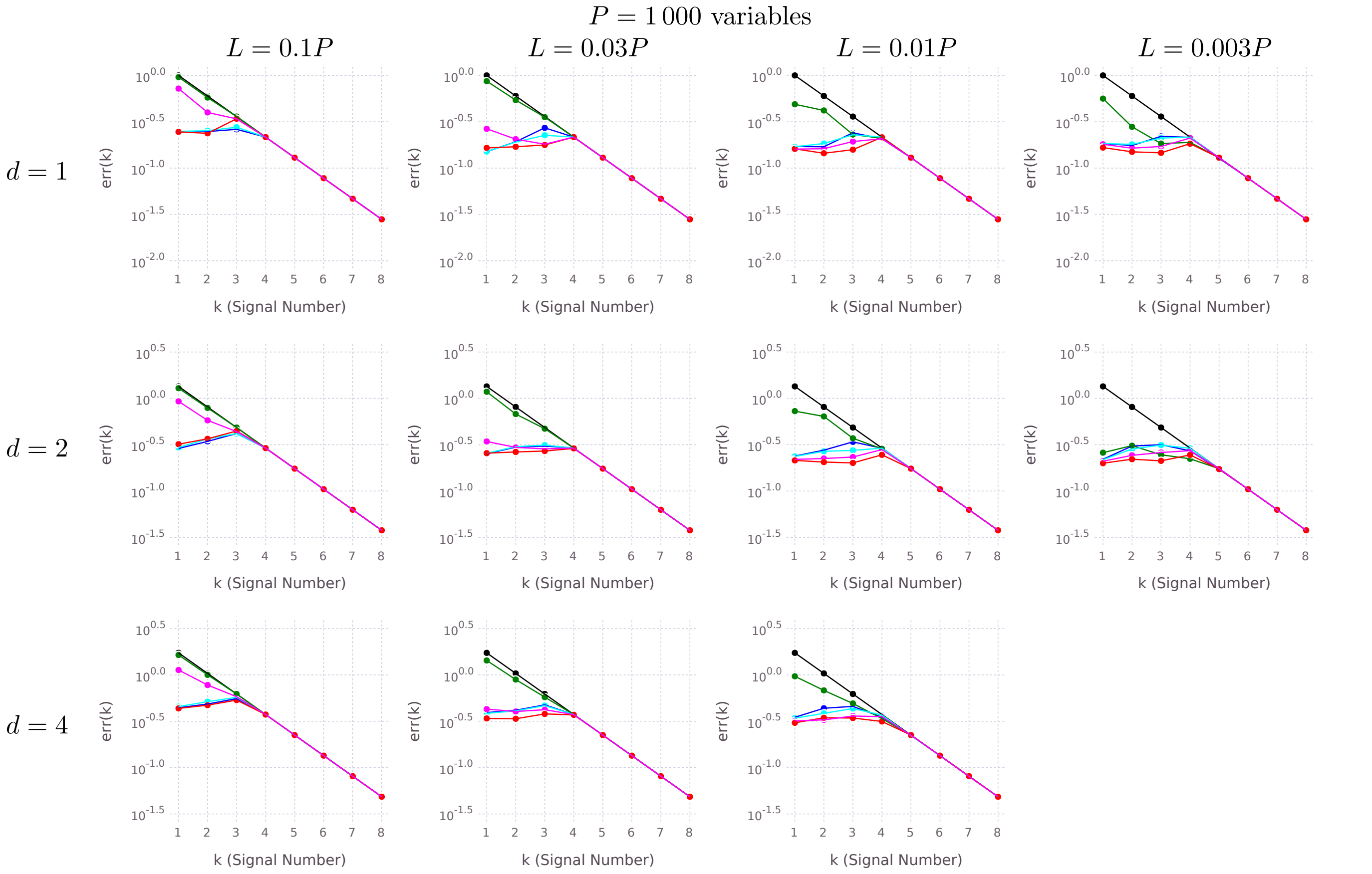

Supplementary Figure 5: The reconstruction error, $\mathrm{err}(k)$, is shown for different conditions. The signal strength $\|Y_k\|_F$ (black) is shown for scale. The methods are: SVD (blue), SMSSVD (red) and SPC (green, magenta, cyan) with decreasing degree of sparsity (regularization parameters $c = 0.04\sqrt{P}$, $c = 0.12\sqrt{P}$ and $c = 0.36\sqrt{P}$ respectively). No errors larger than the signal strength are displayed as that indicates that a different signal has been found.
